# Supplementary material for: A Scoping Review of the Evidence on Prevalence of Feline Upper Respiratory Tract Infections and Associated Risk Factors
Source: Vet Sci. 2024 May 22;11(6):232. doi: 10.3390/vetsci11060232 (PMC11209048; doi:10.3390/vetsci11060232)
Supplement: Supplementary file 1 [file vetsci-11-00232-s001.zip › vetsci-2973943-supplementary.pdf]

Supplementary Table S1: Pre-defined criteria for each field for data extraction

**Article No.**	
**Title**	
**Author**	
**Year**	
**Location (Country)**	Descriptive: Country where study conducted, as mentioned in article (not where it was published or where authors are from)
**Europe**	Binary: based on the country mentioned above (1/0)
**North America**	Binary: based on the country mentioned above (1/0)
**South America**	Binary: based on the country mentioned above (1/0)
**Asia**	Binary: based on the country mentioned above (1/0)
**UK**	Binary: based on the country mentioned above (1/0)
**Trans-Tasman**	Binary: based on the country mentioned above (1/0)
**Time Period**	Descriptive: Exact time range as mentioned in article
**Year Range (Start of Study)**	Factor: Decade range, using the **start date** of study (1971-1980, 1981-1990, etc.up to 2011-2020).Example if a study ran from September 2019 to June 2021, then it gets 2011-2020 as its Year Range.
**Length of Study (in months, not including last month)**	Number: Length calculated in months, including start month and excluding the last month. Example if study goes from June 18 - August 18, that is taken as length of 2 months.
**Type of Study(1)**	Descriptive: Primary Objective of Study (Example: Prevalence, Diagnostic Evaluation, Vaccine Efficacy, etc)
**Type of Study (2)**	Descriptive: Secondary Objective of Study
**Test for FHV-1**	Binary: Has this pathogen been tested for? (1/0)
**Test for FCV**	Binary: Has this pathogen been tested for? (1/0)
**Test for Bb**	Binary: Has this pathogen been tested for? (1/0)
**Test for M.felis**	Binary: Has this pathogen been tested for? (1/0)
**Test for C.Felis**	Binary: Has this pathogen been tested for? (1/0)
**Multiple pathogens**	Number: Total number of pathogens this study tested for
**Dx method-PCR**	Binary: Was this method used as a diagnostic? (1/0)
**Dx method-VI**	Binary: Was this method used as a diagnostic? (1/0)
**Dx method- BI**	Binary: Was this method used as a diagnostic? (1/0)
**Dx method-ELISA**	Binary: Was this method used as a diagnostic? (1/0)
**Dx method-Other**	Binary: Was another method used as a diagnostic? (1/0)
**Other Dx Method**	Descriptive: What other methods were used?
**Sample type-Oropharyngeal Cytobrush**	Binary: Was this kind of sample taken? (1/0)
**Sample type-Oropharyngeal Swab**	Binary: Was this kind of sample taken? (1/0)
**Sample type- Nasal Swab**	Binary: Was this kind of sample taken? (1/0)
**Sample type-Conjunctival Swab**	Binary: Was this kind of sample taken? (1/0)

**Sample type- Blood**	Binary: Was this kind of sample taken? (1/0)
**Sample type- Clinical Signs**	Binary: Were clinical signs used for a cat flu diagnosis? (1/0)
**Sample type- Other sample**	Descriptive: What other samples were taken?
**Sampe Size**	Number: Total animals/clinics included in study
**Cohort- Shelter**	Binary: Type of population tested (1/0)
**Cohort- Owned**	Binary: Type of population tested (1/0)
**Cohort- Other**	Binary: Other populations, like SPF colonies, breeding colonies, strays, ferals (1/0)
**cohort- combined**	Number: Number of cohorts included in study
**Healthy**	Binary: Were symptomatic (cat flu signs) animals included? (1/0)
**Symptomatic**	Binary: Were asymptomatic/healthy animals included? (1/0)
**H+S**	Number: Number of cohorts by clinical status included in study (1 or 2 or NA if not mentioned)
**Risk Factors**	Descriptive: Types of risk factors evaluated
**Risk Factor- Age**	Binary: Was Age evaluated as a risk factor? (1/0)
**Risk Factor- LOS**	Binary: Was LOS (length of Stay in Shelter) evaluated as a risk factor? (1/0)
**Risk Factor- Clinical Signs**	Binary: Were Clinical Signs evaluated as a risk factor? (1/0)
**Prevalence- FHV-1**	Number: Total prevalence as calculated from clinical signs (URI= upper respiratory infection)
**Prevalence- FCV**	Number: Total prevalence for this pathogen (often needs to be manually calculated from various statistics presented in study)
**Prevalence- Bb**	Number: Total prevalence for this pathogen (often needs to be manually calculated from various statistics presented in study)
**Prevalence M.felis**	Number: Total prevalence for this pathogen (often needs to be manually calculated from various statistics presented in study)
**Prevalence- C.felis**	Number: Total prevalence for this pathogen (often needs to be manually calculated from various statistics presented in study)
**Prevalence- URI**	Number: Total prevalence for this pathogen (often needs to be manually calculated from various statistics presented in study)
**Prevalence- Co-infection?**	Number: Prevalence of co-infections (if tested for/reported)
**CI recorded**	Binary: Was Confidence Interval reported for prevalence? (1/0)

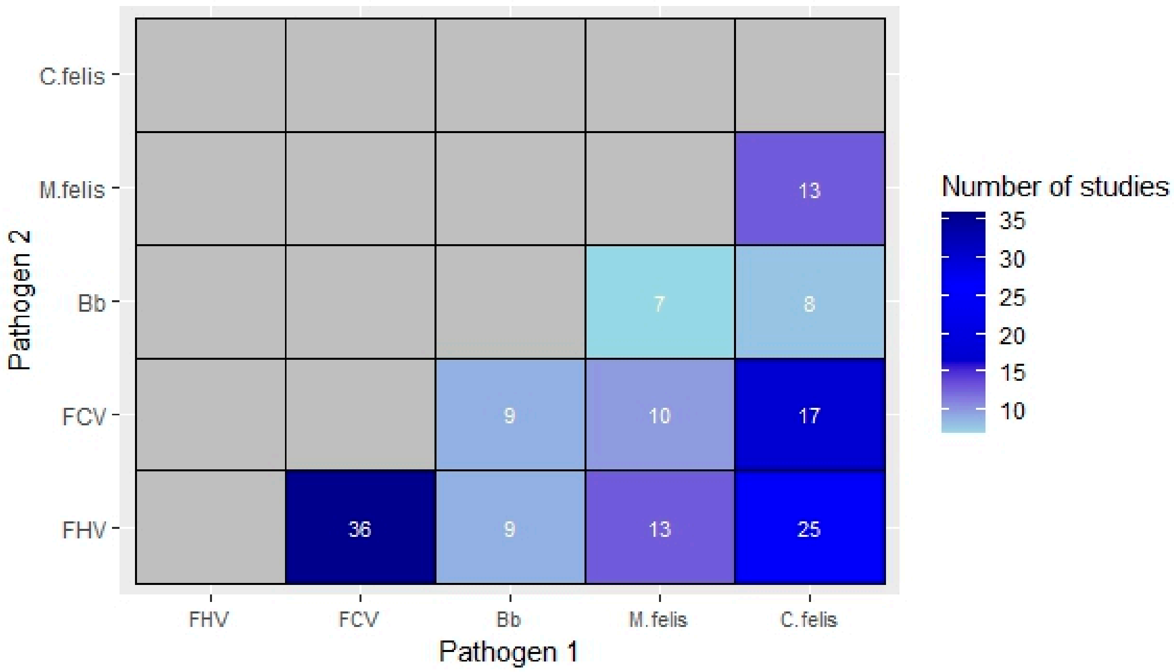

Supplementary Figure S1: Pairwise plot of pathogens tested together by studies
